# Individual patient data network meta-analysis using either restricted mean survival time difference or hazard ratios: is there a difference? A case study on locoregionally advanced nasopharyngeal carcinomas

**DOI:** 10.1186/s13643-019-0984-x

**Published:** 2019-04-15

**Authors:** C. Petit, P. Blanchard, JP. Pignon, B. Lueza

**Affiliations:** 1 Gustave Roussy, Service de Biostatistiques et d’Épidémiologie and Ligue Nationale Contre le Cancer Meta-Analysis Platform, Université Paris-Saclay, F-94805 Villejuif, France; 2 Centre for Research in Epidemiology and Population Health, INSERM U1018, Paris-Saclay University, Villejuif, France; 3 Department of Radiation Oncology, Gustave Roussy, Université Paris-Saclay, F-94805 Villejuif, France

**Keywords:** Hazard ratio, Survival analysis, Restricted mean survival time difference, Network meta-analysis, Nasopharyngeal carcinoma

## Abstract

**Background:**

This study aimed at applying the restricted mean survival time difference (rmstD) as an absolute outcome measure in a network meta-analysis and comparing the results with those obtained using hazard ratios (HR) from the individual patient data (IPD) network meta-analysis (NMA) on the role of chemotherapy for nasopharyngeal carcinoma (NPC) recently published by the MAC-NPC collaborative group (Meta-Analysis of Chemotherapy [CT] in NPC).

**Patients and methods:**

Twenty trials (5144 patients) comparing radiotherapy (RT) with or without CT in non-metastatic NPC were included. Treatments were grouped in seven categories: RT alone (RT), induction CT followed by RT (IC-RT), RT followed by adjuvant CT (RT-AC), IC followed by RT followed by AC (IC-RT-AC), concomitant chemoradiotherapy (CRT), IC followed by CRT (IC-CRT), and CRT followed by AC (CRT-AC). The primary endpoint was overall survival (OS); secondary endpoints were progression-free survival and locoregional control. The rmstD was estimated at t* = 10 years in each trial. Random-effect frequentist NMA models were applied. *P* score was used to rank treatments. Heterogeneity and inconsistency were evaluated.

**Results:**

The three treatments that had the highest effect on OS with rmstD were CRT-AC, IC-CRT, and CRT (respective *P* scores of 92%, 72%, and 64%) compared to CRT-AC, CRT, and IC-CRT when using HR (respective *P* scores of 96%, 71%, and 63%). Of the 32 HR and rmstD analyzed, 5 had a different interpretation, 3 with a direction change (different direction of treatment effect) and 2 with a change in significance (same direction but a change in statistical significance). Results for secondary endpoints were overall in agreement.

**Conclusion:**

The use of either HR or rmstD impacts the results of NMA. Given the sensitivity of HR to non-proportional hazards, this finding could have implications in terms of meta-analysis methodology.

**Electronic supplementary material:**

The online version of this article (10.1186/s13643-019-0984-x) contains supplementary material, which is available to authorized users.

## Introduction

Network meta-analysis (NMA), also known as mixed treatment comparisons, is a statistical method that deals with conditions where multiple treatments have been investigated that have not all been compared pairwise [1]. NMA permits the evaluation of all possible pairwise comparisons based on direct and indirect evidence and allows ranking the different treatments according to their relative efficacies [2]. Similarly to pairwise meta-analysis, NMA uses logarithms (log) of hazard ratios (HR) as input data and outcome measure for survival analysis. However, if the treatment effect varies over time, the proportional hazards assumption might be violated and the HR might thus be considered inaccurate.

Restricted mean survival time (RMST) is an alternative outcome measure that is increasingly used [3–5]. RMST is defined as the mean survival time up to a prespecified time and corresponds graphically to the area under the survival curve. To compare two treatments, the difference of RMST is used (rmstD); it can be expressed as the number of life years gained with the treatment. It is also called life expectancy difference [6]. The use of the rmstD over or in addition to the HR has been advocated in the literature [7, 8]. Firstly, contrary to HR, rmstD remains valid even when the proportional hazards assumption is not respected. Secondly, rmstD is an absolute outcome which depends both on the baseline hazard and on the relative treatment effect, as opposed the HR which solely reflects the relative treatment effect. Also, the interpretation of the treatment difference on the time scale is considered easier, especially from a clinical perspective.

The rmstD has already been empirically compared to HR by Trinquart et al. through 54 randomized controlled trials [9]. The rmstD has also been applied in the context of an individual patient data (IPD) meta-analysis [10–13] but never in the context of NMA. The use of rmstD in oncology has again been recently promoted in a paper by A’Hern [4] as a complementary outcome measure to the HR. The IPD NMA from the MAC-NPC collaborative group (Meta-Analysis of Chemotherapy [CT] in nasopharyngeal carcinoma [NPC]) has been recently published using HR as an outcome measure [14]. The aim of the present study was to apply the rmstD as an absolute measure in a network meta-analysis and to compare the results with those obtained with HR.

## Methods

### MAC-NPC database and endpoints definition

A detailed statistical protocol was written before NMA analysis with rmstD and is available here: https://www.gustaveroussy.fr/sites/default/files/restricted-mean-survival-time-nma-protocol.pdf. The MAC-NPC is an IPD meta-analysis that comprises most randomized trials conducted up to Dec 31, 2010, evaluating the effect of adding chemotherapy to local treatment in patients with non-metastatic NPC. The inclusion criteria, trial search, trial flow chart, data collection, and checking have been detailed in previous publication along with the results of the standard meta-analysis [15]. The primary endpoint was overall survival (OS), defined as the time from randomization until death from any cause. For this work, we chose as secondary endpoints, for exploratory analyses, progression-free survival (PFS) and locoregional control (LRC) since PFS has earlier events than OS and LRC has the fewest events. PFS was defined as the time from randomization to first progression (locoregional or distant) or death from any cause. Patients with a distant failure as a first event were censored for locoregional failure, thus not taking into account competing risks.

### Difference in restricted mean survival time

Mean survival time should be restricted to a specified time t*. The rmstD(t*) corresponds graphically to the area between the two Kaplan-Meier survival curves of the compared treatments up to t*. For the estimation of the rmstD(t*), we selected t* = 10 years for the primary analysis and t* = 5 years for a sensitivity analysis, as these were the two time points of clinical interest in the first update of MAC-NPC [15]. Because the median follow-up of trials was 7.4 years, the majority of the trials included in the NMA had a follow-up long enough using a t* of 10 years. When the latest event in a group of treatment occurred before t*, an extrapolation until t* was performed using the method proposed by Brown et al. [16]. No trial needed extrapolation when the time horizon t* was set at 5 years. Of note, in case of t* = 5 years, we censored the follow-up for HR estimation at 5 years so that both treatment effect measures—rmstD and HR—are more comparable.

### Statistical methods for pairwise meta-analysis

In each pairwise meta-analysis, we used the pooled Kaplan-Meier method with DerSimonian-Laird random effect to estimate the rmstD(t*). This method consists in aggregating trial-specific rmstD(t*) which are estimated for each trial as the area between the two Kaplan-Meier survival curves. A previous study comparing different methods to estimate the rmstD from IPD meta-analysis showed that this method was the best compromise in terms of bias and variance [17]. Heterogeneity was quantified using the *I*^2^, which represents the proportion of total variation in study estimates that is due to heterogeneity [18].

### Statistical methods for network meta-analysis

A two-step method was used. The first step was to compute rmstD(t*) for each trial using individual patient data. The network meta-analysis was then performed using a frequentist approach using as input data for each trial comparison the two treatments compared, the rmstD, and its variance. Based on the Grambsch-Therneau test, the proportional hazards assumption was checked for each trial comparisons [19] and for each pairwise meta-analysis [10].

To limit the number of tests for both heterogeneity and inconsistency, Rücker et al. have proposed a global test, called the *Q* test [20]. This test is a generalization of Cochran’s test that is used to assess heterogeneity in conventional meta-analysis. The *Q* statistic is the sum of a statistic for heterogeneity (within designs) and a statistic for inconsistency (between designs). Inconsistency can be defined as the variability of treatment effect between direct and indirect comparisons at the meta-analytic-level. The protocol stated that a random effects model had to be used to aggregate rmstD(t*), even without heterogeneity (*P* value > .1). This choice was made based on a previous work which showed that a fixed effect model underestimates the variance of the overall rmstD(t*) [17]. In case of inconsistency, the trials responsible for inconsistency were determined by comparing direct and indirect estimates and trial forest plots within the inconsistent closed loop; the effect of trial removal on the network consistency and estimation could therefore be investigated.

The treatments were ranked using the *P* score, which measures the mean extent of certainty that a treatment is better than the competing treatments [21]. *P* score would be 1 when a treatment is certain to be the best and 0 when a treatment is certain to be the worst.

This work was performed in accordance with the NMA guidelines [22]. *P* values < .05 were considered significant for the difference between treatments. All analyses were performed using the R software (version 3.4.0) and the R package *netmeta* [23].

## Results

### Description of the network and patients

Details concerning the trials included and the network have been previously published [14, 15]. In summary, the network consists of 20 trials and 5144 patients [24–42]. Because of a factorial design in two trials, these 20 trials were split into 26 trial comparisons. There were seven different treatments: radiotherapy (RT) alone, which was used as the reference category; induction chemotherapy (IC) followed by RT (IC-RT); RT followed by adjuvant chemotherapy (AC) (RT-AC); IC followed by RT followed by AC (IC-RT-AC); concomitant chemoradiotherapy (CRT); IC followed by CRT (IC-CRT); and CRT followed by AC (CRT-AC). Only IC-CRT was not directly compared with RT. The network is represented in Additional file 1: Figure S1. Median follow-up based on individual patient data (interquartile range) was 7.4 years (4.9 to 10.6). Proportional hazards assumption was verified in each pairwise meta-analysis. For each of the 26 trial comparisons, treatment effect estimates based on HR and rmstD and proportional hazard assumption test for OS are presented in Table 1. According to the Grambsch-Therneau test, the proportional hazards assumption was not verified at the 5% significance level for two trials for OS (NPC008 [32] and Guangzhou 2002-02 [33]). Further details can be found in the original publication of the NMA [14]. Cumulative incidence curves for OS, PFS, and LRC events are presented in Additional file 2: Figure S2.

**Table 1:** Summary table for overall survival with HR and rmstD at t* = 5 years and 10 years and their respective confidence intervals for each trial comparison of the network meta-analysis

Trial comparison	Treatment comparison	HR	CI 95%	*p* value test for non-proportionality	rmstD (m) t* = 5 years	CI 95%	rmstD (m) t* = 10 years	CI 95%
AOCOA	IC-RT vs. RT	0.99	[0.68; 1.44]	0.60	0.66	[− 4.05; 5.36]	− 0.39	[− 12.15; 11.36]
VUMCA-89	IC-RT vs. RT	1.00	[0.75; 1.33]	0.49	0.56	[− 4.34; 5.45]	0.73	[− 10.06; 11.52]
Japan-91	IC-RT vs. RT	0.77	[0.40; 1.46]	0.27	0.91	[− 6.21; 8.02]	9.44*	[− 10.19; 29.07]
PWHQEH-94	CRT vs. RT	0.81	[0.61; 1.07]	0.11	4.24	[0.69; 7.79]	9.11	[0.14; 18.08]
QMH-95Conc^‡^	CRT vs. RT	1.00	[0.57; 1.75]	0.75	1.18	[− 4.18; 6.54]	− 0.30	[− 14.83; 14.24]
Guangzhou 2001	CRT vs. RT	0.54	[0.31; 0.93]	0.080	7.90	[1.98; 13.82]	17.94	[2.53; 33.35]
Guangzhou 2003	CRT vs. RT	0.34	[0.18; 0.66]	0.91	1.95	[0.06; 3.84]	10.29*	[3.71; 16.87]
INT-0099	CRT-AC vs. RT	0.50	[0.36; 0.71]	0.11	11.92	[6.13; 17.71]	27.08	[14.27; 39.89]
QMH-95Comp5^‡^^	CRT-AC vs. RT	0.65	[0.36; 1.19]	0.79	1.79	[− 3.67; 7.25]	8.02	[− 6.05; 22.08]
SQNP01	CRT-AC vs. RT	0.68	[0.48; 0.96]	0.52	5.30	[0.45; 10.14]	14.30	[2.90; 25.70]
NPC-9901	CRT-AC vs. RT	0.73	[0.54; 0.99]	0.24	1.50	[− 2.37; 5.37]	6.58	[− 2.75; 15.91]
NPC-9902CF	CRT-AC vs. RT	0.97	[0.52; 1.82]	0.53	1.67	[− 4.55; 7.88]	2.94	[− 13.50; 19.37]
NPC-9902AF	CRT-AC vs. RT	0.50	[0.28; 0.90]	0.76	6.37	[0.67; 12.07]	16.73	[1.48; 31.97]
Guangzhou 2002-01	CRT-AC vs. RT	0.69	[0.48; 0.99]	0.16	4.68	[0.97; 8.38]	7.21*	[− 3.21; 17.64]
TCOG-94	RT-AC vs. RT	0.95	[0.65; 1.40]	0.24	− 1.52	[− 7.94; 4.90]	1.57	[−13.01; 16.15]
QMH-95Adj^‡^	RT-AC vs. RT	1.07	[0.61; 1.89]	0.30	− 3.24	[− 9.54; 3.05]	− 6.55	[− 22.30; 9.21]
VUMCA-95	IC-CRT vs. IC-RT	0.89	[0.69; 1.16]	0.58	0.67	[− 3.05; 4.40]	4.33*	[− 5.07; 13.73]
Guangzhou 2002-02	IC-CRT vs. IC-RT	0.95	[0.69; 1.30]	0.027	− 1.63	[− 4.83; 1.58]	0.60*	[− 7.68; 8.87]
NPC008	CRT vs. IC-CRT	1.57	[0.72; 3.41]	0.030	− 9.82	[− 17.31; − 2.34]	− 18.16*	[− 37.67; 1.36]
HeCOG	CRT vs. IC-CRT	1.01	[0.60; 1.68]	0.48	− 0.03	[− 6.98; 6.92]	− 0.91*	[− 16.86; 15.04]
QMH-95Adj+^‡^	CRT-AC vs. CRT	0.66	[0.36; 1.19]	0.99	0.61	[− 4.28; 5.51]	8.32	[− 5.36; 21.99]
Guangzhou 2006	CRT-AC vs. CRT	0.79	[0.47; 1.30]	0.85	1.53	[− 0.84; 3.89]	6.30*	[− 3.20; 15.80]
QMH-95Conc+^‡^	RT-AC vs. CRT-AC	1.59	[0.87; 2.91]	0.24	− 5.03	[− 10.94; 0.88]	− 14.56	[− 29.53; 0.40]
QMH-95Comp6^‡^^	RT-AC vs. CRT	1.07	[0.61; 1.87]	0.11	− 4.42	[− 10.23; 1.40]	− 6.25	[− 21.66; 9.16]
PWH-88	IC-RT-AC vs. RT	1.30	[0.62; 2.73]	0.78	− 3.28	[− 12.56; 6.01]	− 9.17*	[− 32.69; 14.36]
Shanghai 2004	IC-RT-AC vs. CRT-AC	1.15	[0.61; 1.81]	0.80	− 0.32	[− 2.41; 1.78]	− 3.57*	[− 12.52; 5.39]

*CI* confidence intervals; *HR* hazard ratio; *m* months; *rmstD* restricted mean survival time difference; *PWH* Prince of Wales Hospital; *AOCOA* Asian-Oceanian Clinical Oncology Association; *VUMCA* International Nasopharynx Cancer Study Group (cavum); *PWHQEH* Prince of Wales Hospital, Queen Elizabeth Hospital; *INT-0099* SWOG (Southwest Oncology Group)-coordinated Intergroup trial, also known as SWOG 8892; *QMH* Queen Mary Hospital; *SQNP* Singapore Naso-Pharynx; *NPC* nasopharyngeal carcinoma; *CF* conventional fractionation; *AF* accelerated fractionation; *TCOG* Taiwan Cooperative Oncology Group; *HeCOG* Hellenic Cooperative Oncology Group; *RT* radiotherapy; *IC* induction chemotherapy; *CRT* concomitant chemoradiotherapy; *AC* adjuvant chemotherapy

^‡^QMH-95 trial, 2 × 2 design, considered as a multi-arm trial, and split into six comparisons

*Extrapolation performed until 10 years using the Brown et al. method [16]

^^^Comparison estimated using individual patient data, required for computation of multi-arms trials

### Restricted mean survival time difference

For each of the 26 trial comparisons, the estimates RMST for each arm and their difference (rmstD), at t* = 5 years and t* = 10 years, are displayed in (Additional file 3: Table S1). For example, regarding to VUMCA-89 trial [24], where IC-RT was compared to RT, the estimated RMST(t* = 10 years) were respectively 64.70 and 63.96 months and thus the rmstD(t* = 10 years) was 0.73 months, in favor of IC-RT, and its 95% confidence interval (CI) was (− 10.06 to 11.52) not significant (contains zero). In other words, IC-RT extended the life expectancy during the first 10 years of follow-up by a non-significant 0.73 months as compared to RT.

Network meta-analysis (NMA) using rmstD showed that the three treatments that had the highest effect on OS with rmstD(t* = 10 years) were CRT-AC, IC-CRT, and CRT with respective *P* scores of 0.92, 0.72, and 0.64, respectively. There was no significant heterogeneity (*I*^2^ = 14.7%, *P* = .29) or inconsistency (*P* = .33). The rmstD(t* = 10 years) and their 95% CI on the basis of the NMA for each pairwise comparison are presented in the lower left triangle of the league table (Table 2). Compared with RT alone, the rmstD(t* = 10 years) (95% CIs) on OS for CRT-AC, IC-CRT and, CRT respectively, were 11.89 months (7.40 to 16.38), 8.71 months (0.26 to 17.16), and 7.67 (2.91 to 12.43). The rmstD(t* = 10 years) (95% CIs) of CRT-AC compared with IC-CRT or CRT showed no significant differences, with respective values of 3.18 (− 6.18 to 12.53) and 4.22 (− 1.50 to 9.93) months.

**Table 2:**
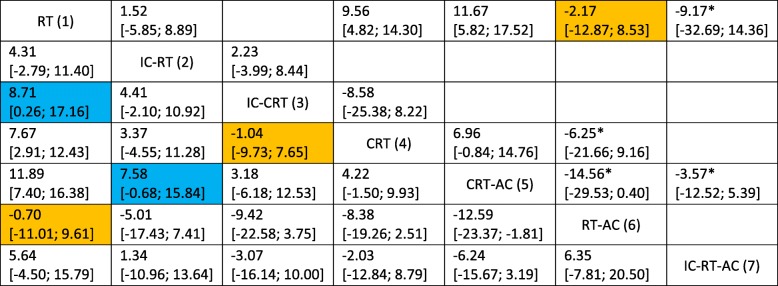
League table presenting the results with difference in restricted mean survival time (in months) of the network meta-analysis (random effects, lower triangle) and of the conventional meta-analysis (random effects, upper triangle) for overall survival at t* = 10 years

*I*^2^ = 14.7%, heterogeneity (within design) *p* = 0.29, inconsistency (between designs) *p* = 0.33. Individual trial (comparison) rmstD are given in Table 1. As a convention, the cells contain the difference in restricted mean survival time in months (rmstD; 95% confidence interval) of the treatment with the higher number compared to the treatment with the lower number. For example, the cell that joins treatments 4 (CRT) and 5 (CRT-AC) gives the rmstD of treatment 5 vs. 4 (CRT-AC vs. CRT)

Same direction of treatment effect but difference in significance between HR and rmstD

Different direction of treatment effect but both HR and rmstD are not significant

*AC* adjuvant chemotherapy, *CRT* concomitant chemoradiotherapy, *HR* hazard ratio, *IC* induction chemotherapy, *rmstD* restricted mean survival time difference, *RT* radiotherapy

*Comparison with only one trial

### Comparison of results obtained using rmstD and HR

Among the estimated HR and rmstD(t* = 10 years) on OS for each of the 26 trial comparisons (Table 1), 23 had the same interpretation (direction of treatment effect and significance or not): for 5 comparisons, both rmstD and HR were significant (Additional file 4: Figure S3); for 18 comparisons, both rmstD and HR were not significant, including the two trials with non-proportional hazards (NPC008 [32] and Guangzhou 2002-02 [33]). One comparison had a different direction of treatment effect (HR < 1 and rmstD < 0) but both HR and rmstD were not significant (AOCOA [40]); throughout this paper, this change will be named a direction change. Three comparisons had the same direction of treatment effect but changed in significance, namely a change in significance. For two of them, rmstD was not significant whereas HR was significant (NPC-9901 [29] and Guangzhou 2002-01 [34]); for the remaining one, rmstD was significant whereas HR was not significant (PWHQEH-94 [42]).

**Fig. 1:**
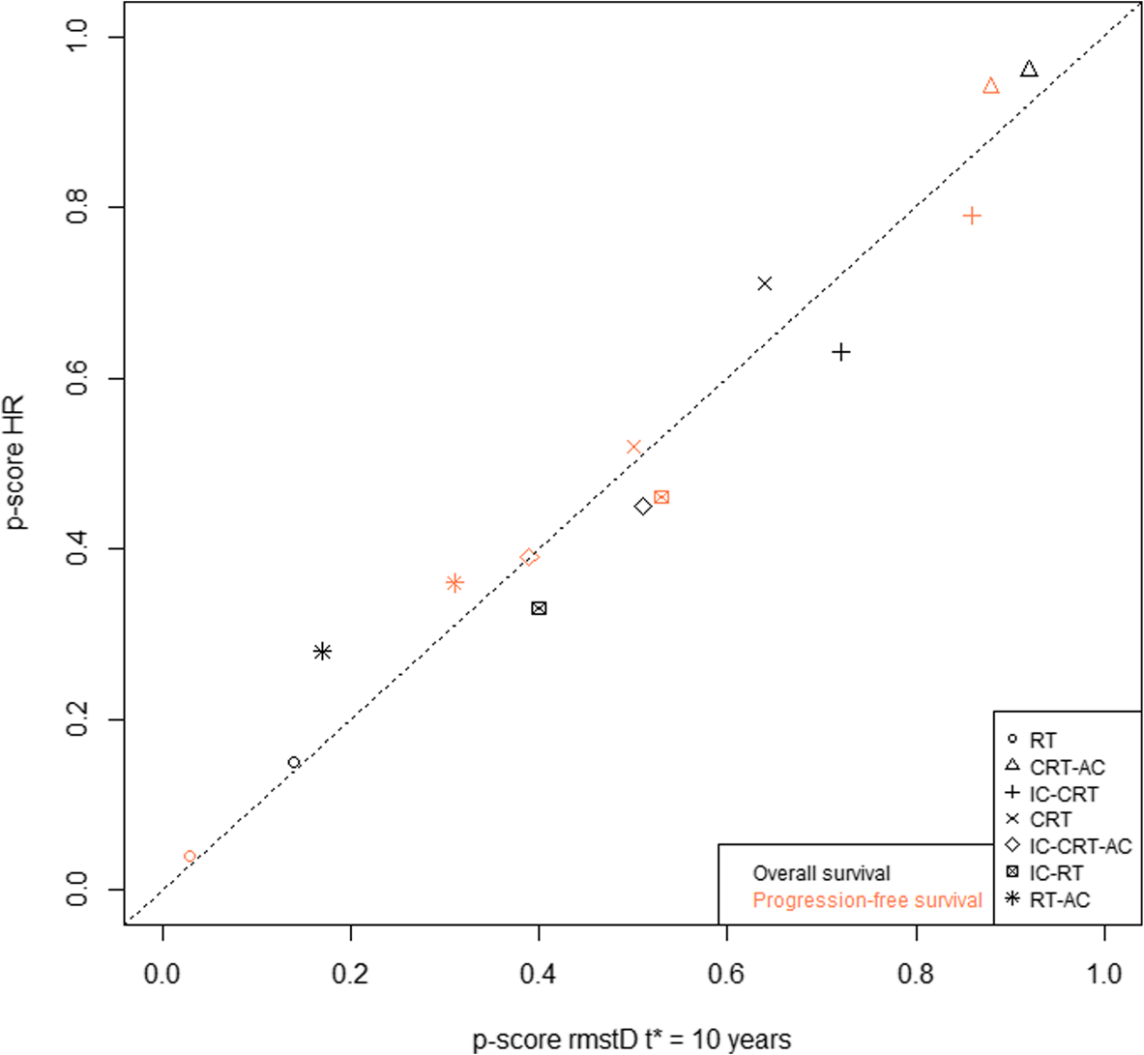
*P* scores for overall survival and progression-free survival according to the network meta-analysis with hazard ratios and restricted mean survival time difference at t* = 10 years. AC adjuvant chemotherapy, CRT concomitant chemoradiotherapy, HR hazard ratio, IC induction chemotherapy, rmstD restricted mean survival time difference, RT radiotherapy

**Fig. 2:**
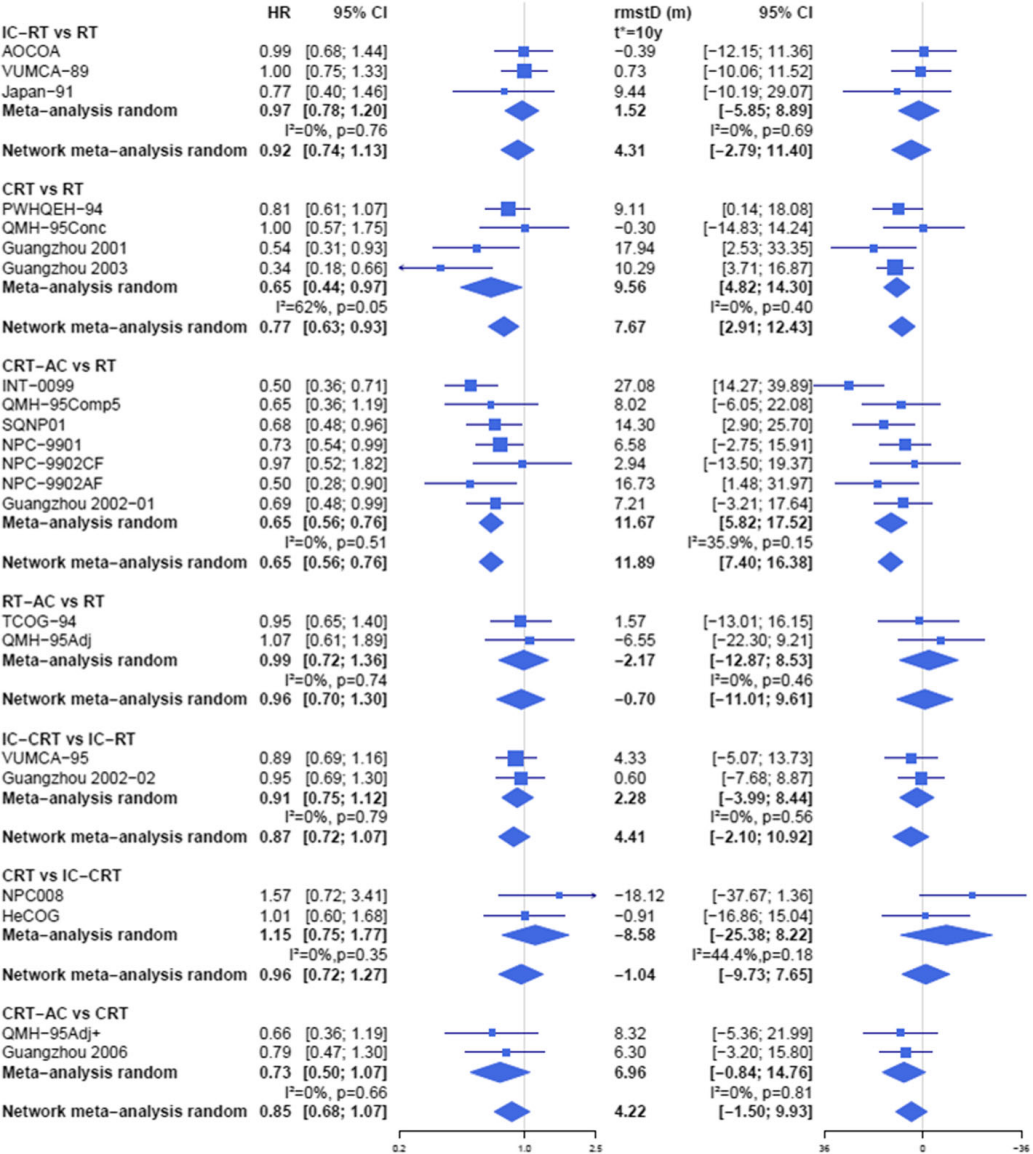
Forest plot for overall survival with hazard ratios (on the left) and restricted mean survival time difference at t* = 10 years (on the right) showing results from direct comparisons (random effects meta-analysis) and network meta-analysis. Only comparisons involving more than one trial are presented. Within each meta-analysis, *I*^2^ represents the proportion of total variation in study estimates that is due to heterogeneity [18], *p* represents the *p* value for the heterogeneity statistic *Q*. AC adjuvant chemotherapy, CRT concomitant chemoradiotherapy, HR hazard ratio, IC induction chemotherapy, rmstD restricted mean survival time difference, RT radiotherapy. The scale of the rmstD was inverted with a negative value on the right, in order to allow visual comparisons with HR. Trial names are defined in Table 1 footnote

In the NMA based on HR, the three treatments that had the highest effect on OS were CRT-AC, CRT, and IC-CRT, with respective *P* scores of 0.96, 0.71, and 0.63, respectively. We observed a reversal of the ranking of the treatments ranked second and third compared to rmstD(t* = 10 years). For both endpoints, OS and PFS, *P* scores obtained using rmstD(t* = 10 years) and HRs are given in Fig. 1 for the seven treatments. In the network, and especially where there was direct information, the HR and rmstD measures were in agreement overall (Fig. 2). Indeed, on the 32 HR and rmstD(t* = 10 years) obtained in the league tables (21 for the NMA and 11 for conventional MA, Table 2 and Additional file 5: Table S2), 27 had the same interpretation (treatment effect and significance). However, three had a direction change but close to the null treatment effect (HR = 1 and rmstD = 0) and two had a change in significance. Those two pairwise comparisons had only indirect information. The first one concerned the comparison of IC-CRT with RT, where HR was non-significant, 0.80 (95% CI, 0.62 to 1.04), while rmstD(t* = 10 years) was significantly in favor of IC-CRT, with a value of 8.71 months (95% CI, 0.26 to 17.16). The second one concerned the comparison of CRT-AC with IC-RT, where HR was significantly in favor of CRT-AC, 0.71 (95% CI, 0.55 to 0.92), while rmstD(t* = 10 years) was not, 7.58 months (95% CI, − 0.68 to 15.84). Detailed analysis of those two differences is given in Additional file 6: Text S1.

The reversal in the ranking (IC-CRT becomes better than CRT with rmstD) is partially explained by the change in significance, in favor of IC-CRT, in the comparison of IC-CRT with RT but also because of a direction change in the comparison of CRT with IC-CRT, still in favor of IC-CRT, where HR was 0.96 (95% CI, 0.72 to 1.27) while rmstD(t* = 10 years) was − 1.04 months (95% CI, − 9.73 to 7.65).

### Sensitivity analyses

Three sensitivity analyses for OS have been performed, one with another estimation of the rmstD at t* = 5 years and compared with HR censored at 5 years (Additional file 7: Table S3), a second after the exclusion of trials with non-proportional hazards (Additional file 8: Table S4), and a third one after the exclusion of Guangzhou 2003 [35], which had the largest difference between rmstD and HR estimation (Fig. 2). This large difference for this trial, with follow-up similar to the other trials, may be explained by a lower baseline hazard as compared to the other trials. Thus, the treatment effect expressed on the relative scale is high with a HR of 0.34 and led to an absolute rmstD(t* = 10 years) of 10.29 months as compared for instance to a HR of 0.54 and rmstD(t* = 10 years) of 17.94 months for Guangzhou 2001 [31] with higher baseline hazard (Fig. 2). The two first sensitivity analyses were planned and the third one was exploratory. In these three analyses, CRT-AC remained ranked first as in the main analysis. The first sensitivity analysis was done at 5 years with HR censored to compare with rmstD(t* = 5 years), a time horizon at which we did not need to extrapolate. On the 32 HR censored and rmstD(t* = 5 years) obtained in the league table (Additional file 7: Table S3), 28 had the same interpretation and four had a direction change, with both HR and rmstD not significant. CRT-AC remains ranked first but there were many changes afterwards in the ranking with the *P* score. In the second sensitivity analysis, after exclusion of trials with non-proportional hazards, the ranking for rmstD was the same for the first four modalities of treatment as compared to the ranking with HR after exclusion of trials (Additional file 8: Table S4) and was identical to the ranking obtained for OS with HR on all trials (Table 3). The analysis after the exclusion of Guangzhou 2003 [35] gave results similar to the main analysis.

**Table 3:** Summary table of network meta-analysis results with the difference of restricted mean survival time and the hazard ratio for the six treatments compared with radiotherapy alone and the three efficacy endpoints

Treatment data	Overall survival		Progression-free survival		Locoregional control	
20 trials, 5144 patients	26 comparisons, 2070 events		26 comparisons, 2489 events		26 comparisons, 915 events	
	rmstD(t*=10 years) (m)	Hazard ratio	rmstD(t*=10 years) (m)	Hazard ratio	rmstD(t*=10 years) (m)	Hazard ratio
*P* value heterogeneity/inconsistency	0.28	0.39	0.47	0.57	0.50	0.66
P value heterogeneity	0.29	0.30	0.22	0.24	0.26	0.35
P value inconsistency	0.33	0.54	0.87	0.96	0.84	0.92
RT*						
*P* score	0.14	0.15	0.03	0.04	0.03	0.03
CRT-AC	11.89 [7.40; 16.83]	0.65 [0.65; 0.76]	16.10 [11.70; 20.50]	0.62 [0.54; 0.71]	8.76 [5.21; 12.31]	0.53 [0.41; 0.68]
*P* score	0.92	0.96	0.88	0.94	0.71	0.82
IC-CRT	8.71 [0.26; 17.16]	0.80 [0.62; 1.04]	16.12 [7.97; 24.27]	0.68 [0.54; 0.85]	5.97 [− 1.75; 13.68]	0.72 [0.51; 1.01]
*P* score	0.72	0.63	0.86	0.79	0.47	0.50
CRT	7.67 [2.91; 12.43]	0.77 [0.63; 0.93]	10.59 [5.91; 15.26]	0.77 [0.65; 0.91]	5.08 [1.49; 8.68]	0.78 [0.58; 1.05]
*P* score	0.64	0.71	0.50	0.52	0.36	0.38
IC-RT-AC	5.64 [− 4.50; 15.79]	0.87 [0.58; 1.31]	8.10 [− 1.88; 18.09]	0.83 [0.58; 1.17]	12.04 [3.40; 20.67]	0.47 [0.27; 0.82]
*P* score	0.51	0.45	0.39	0.39	0.86	0.87
IC-RT	4.31 [− 2.79; 11.40]	0.92 [0.74; 1.13]	11.10 [4.37; 17.80]	0.79 [0.66; 0.93]	5.65 [− 1.36; 12.66]	0.83 [0.64; 1.07]
*P* score	0.40	0.33	0.53	0.46	0.43	0.28
RT-AC	− 0.70 [− 11.01; 9.61]	0.96 [0.70; 1.30]	6.37 [− 4.79; 17.53]	0.84 [0.63; 1.11]	8.76 [− 0.74; 18.27]	0.63 [0.37; 1.06]
*P* score	0.17	0.28	0.31	0.36	0.65	0.63

*m* months, *rmstD* restricted mean survival time difference, *RT* radiotherapy, *IC* induction chemotherapy, *CRT* concomitant chemoradiotherapy, *AC* adjuvant chemotherapy

*Reference treatment

### Secondary endpoints

Results for secondary endpoints are presented in Table 3. The results of PFS (Additional file 9: Table S5, Additional file 2: Figure S2 and Additional file 10: Figure S4) were in agreement with OS and CRT-AC remains ranked first. On the 32 HR and rmstD(t* = 10 years) obtained in the league table, 28 had the same interpretation. Detailed analysis of the differences is given in Additional file 6: Text S2. For LRC, the three best treatments were IC-RT-AC, CRT-AC, and RT-AC with both HR and rmstD (Table 3). There was no increase in the number of discrepancies between HR and rmstD(t* = 10 years) as compared with OS (Additional file 11: Table S6 and Additional file 12: Figure S5). On the 32 HR and rmstD(t* = 10 years) obtained in the league table, 28 had the same interpretation. Detailed analysis of the differences is given in Additional file 6: Text S3. Sensitivity analyses after the exclusion of trials with non-proportional hazards (for PFS: INT-0099 [25], NPC-9902AF [30], Guangzhou 2001 [31], NPC008 [32] and for LRC : VUMCA 89 [24], INT-0099 [25], NPC-9902AF [30]) did not resolve the discrepancies for neither PFS nor LRC.

## Conclusions

The aim of this study was to compare two treatment effects measure, HR and rmstD, and not to identify the best treatment as previously published with HR. So, in our case study, even if treatment effect measures on the basis of HR and rmstD are not interchangeable, the results of the NMA with rmstD and HR were in agreement most of the time. Indeed, on the 32 HR and rmstD(t* = 10 years) obtained in the league table (21 for the NMA and 11 for conventional MA), 27 had the same interpretation for overall survival (direction of treatment effect and significance) and 28 for both progression-free survival and loco-regional control. Overall, it is worth noting that for both the 26 trial comparisons and the 32 network meta-analyses comparisons, the rmstD and the HR never led to both a change in the direction of the treatment effect and a change in significance. In all analyses, the same treatment was ranked first with HR and rmstD, and when there was an inversion in the order of ranking, the treatments concerned were not significantly different.

An important matter when comparing HR and rmstD is the proportional hazards assumption. Indeed, different results at the level of trial comparison can be obtained for trials with non-proportional hazards and for which the HR should not be used [7]. In our study, trials with non-proportional hazards did not have discrepancies between HR and rmstD at t* = 10 years at the trial level but impacted the results obtained at the NMA level. The majority of these discrepancies concerned the direction of the treatment effect. Although these changes were close to the null effect, the direction change had an important effect on the results of the NMA, highlighting the sensitivity of NMA to minor direction changes. The other discrepancies, less frequent, corresponded to a change in significance. These changes in significance were in indirect comparisons in which trials with non-proportional hazards were involved. For OS, the discrepancies disappeared when these trials were removed in a sensitivity analysis. However, for PFS and LRC, this was not the case. This could be explained by the fact that trials with non-proportional hazards were not located in the part of the network where the discrepancies were found. Thus, non-proportional hazards can only partly explain the differences between HR and rmstD.

Another important issue, already pointed out in the previous publications on the use of rmstD(t*), is the choice of the time horizon (t*), especially in the context of MA and NMA as trials often have different follow-up [10, 11, 15]. An extrapolation had to be used for trials where the latest event was before 10 years and thus added information when estimating rmstD as compared to HR. At t* = 10 years, 20 of the trial arms (45.5%) had a latest event before 10 years of follow-up and required extrapolation with a mean time of 1.8 years (IQR, 0.8–2.3 years). Regarding the median follow-up, it was longer than 10 years for 7 of the 20 studies (35.0%) included in the MA. The proportions of the total number of events observed at 10 years were 94.8%, 96.6%, and 99.0% respectively for OS, PFS, and LRC (Additional file 2: Figure S2). When using t* = 5 years, all trials had a follow-up long enough and none required survival extrapolation (Additional file 3: Table S1), but we used censored HR for better comparability which leads to a loss of information. The results obtained at t* = 5 years showed also differences between HR and rmstD. Therefore, extrapolation does not seem to explain the differences and can be used if needed but keeping the extrapolation time relatively short. The most important requirement is to prespecify the time horizon(s) t* at the time of the study design in accordance with clinical interest [8]. Indeed, if survival curves cross—one of the scenarios in which hazards are not proportional and HR estimation will be biased—the choice of the time horizon t* may be critical for the estimation of rmstD, as a treatment may offer a positive benefit in RMST at shorter follow-up times, but a negative one if t* was set at a longer time.

To evaluate the robustness of the NMA with rmstD, we studied two other outcomes than OS. PFS has earlier events and is a strong surrogate for OS [43]. LRC has fewer events which could have increased the uncertainty around the estimation of the treatment effect and the discrepancies. In both cases, the results were overall in agreement and showed that a NMA using rmstD is feasible for different clinical outcomes. Nevertheless, the advantages of rmstD do not overcome the major limitation of the NMA, the use of indirect comparisons which have a lower value than the direct ones.

Our study has several strengths. We used individual patient data to obtain HR and rmstD in order to compare the results of a frequentist NMA with two outcome measure. Therefore, we would be able to conclude with reasonable confidence whether in practice the choice of outcome measure matters to NMA results. We used random effects for both HR and rmstD NMAs to avoid underestimation of the variance of the overall rmstD(t*) [17]. Furthermore, a protocol for our study was written before the start of the analysis with the aim of clearly defining the objectives, design, and methods. Lastly, data on long-term follow-up was available increasing the probability to observe non-proportional hazard.

The present work has limitations. We compared rmstD and HR through a case study under favorable conditions. Indeed, we had no heterogeneity in the NMA, few trials had non-proportional hazards, there was no difference in baseline hazards for our trials (except Guangzhou 2003 [35]), and most of the trials had a long follow-up. We used an extrapolation method developed by Brown et al. [16] which has been previously shown to perform well in a simulation study by Lamb and colleagues [44]. More complex types of extrapolation can be performed through the use of parametric survival models such as the flexible parametric model developed by Royston and Parmar [45]. Of note, a recent methodological paper by Freeman and Carpenter [46] proposed to deal with non-proportional hazards using the Royston-Parmar parametric model in the context of Bayesian IPD NMA. However, this paper used hazard ratios as treatment effect outcome measures and not rmstD. For loco-regional control, we did not use a competing risk model to take into account distant failure. However, in case of a non-negligible number of competing events, the corresponding Kaplan-Meier estimator is biased, and thus, the RMST should be estimated under a competing risk framework. For instance, one could use the methodology developed by Calkins et al. who recently published an extension of RMST to competing risks [47]. Lastly, we focused on the significance of the estimations as the two metrics studied are not directly comparable. However, to avoid too much emphasis on significance, especially given the number of comparisons, confidence intervals should also be taken into account to interpret the results.

As far as we know, our study is the first NMA using the rmstD(t*) as the outcome measure for the treatment effect. But this is not the first publication with other outcome measures for survival data in NMA than HR. Ouwens et al. proposed a method based on Weibull survival curves [48]. It is a more flexible approach where a two-dimensional estimation of the treatment effect based on both shape and scale parameters of parametric survival functions are used. Notably, this method can be used when the proportional hazards assumption is not appropriate. Nonetheless, this model is not yet developed for multiple-arm trials whereas our network contained a four-arm trial [27]. Our results showed that a NMA using rmstD is feasible and reliable.

Currently, in clinical research, the focus is shifting from CRT-AC to IC-CRT or to biomarker-based strategies (NCT02135042). A second update of the MAC-NPC meta-analysis is ongoing. Nevertheless, improving methods used to compare treatments is an important methodological goal. Future research should focus on the differences in outcome measure between HR and rmstD that are related to the proportionality of hazards and the baseline hazards. In addition, we know that the RMST estimates incorporate the number of events and the exposure times whereas the HR estimates and the width of its confidence interval mostly depend on the number of observed events [49]. Simulation studies would be necessary to better understand the ways in which rmstD and HR respond to variations in these determinants.

## Additional files

Additional file 1: **Figure S1.** Graphical representation of the trial network for overall survival. (JPG 51 kb)

Additional file 2: **Figure S2.** Cumulative incidence curves for overall survival, progression free survival and loco-regional control events. (TIF 179 kb)

Additional file 3: **Table S1.** Descriptive table of RMST per study and per arm and the rmstD at t* = 5 years and t* = 10 years for overall survival, expressed in months, and follow-up information. (DOCX 33 kb)

Additional file 4: **Figure S3.** Estimates of treatment effect for overall survival according to hazard ratios and restricted mean survival time difference at t* = 10 years. (TIFF 89 kb)

Additional file 5: **Table S2.** League table presenting the results with hazard ratio of the network meta-analysis (random effects, lower triangle) and of the conventional meta-analysis (random effects, upper triangle) for overall survival. (DOCX 18 kb)

Additional file 6: **Text S1.** Details of differences between restricted mean survival time difference and hazard ratio in the network meta-analysis for overall survival. **Text S2** Details of differences between restricted mean survival time difference and hazard ratio in the network meta-analysis for progression-free survival. **Text S3** Details of differences between restricted mean survival time difference and hazard ratio in the network meta-analysis for loco-regional control. (DOCX 25 kb)

Additional file 7: **Table S3.** League table presenting the results with difference in restricted mean survival time (in month) and hazard ratio with censor at 5 years of the network meta-analysis (random effects, lower triangle) and of the conventional meta-analysis (random effects, upper triangle) for overall survival at t* = 5 years (sensitivity analysis). (DOCX 17 kb)

Additional file 8: **Table S4.** League tables presenting the results with difference in restricted mean survival time (in month) at t* = 10 years and hazard ratio of the network meta-analysis (random effects, lower triangle) and of the conventional meta-analysis (random effects, upper triangle) for overall survival after exclusion of NPC008 and Guangzhou 2002-02 trials which had significant test for non-proportionality (sensitivity analysis). (DOCX 17 kb)

Additional file 9: **Table S5.** League tables presenting the results with restricted mean survival time difference (in month) at t* = 10 years and hazard ratio of the network meta-analysis (random effects, lower triangle) and of the conventional meta-analysis (random effects, upper triangle) for progression-free survival. (DOCX 20 kb)

Additional file 10: **Figure S4.** Forest plot for progression-free survival with hazard ratios (on the left) and restricted mean survival time difference at t* = 10 years (on the right) showing results from direct comparisons (random effects meta-analysis) and network meta-analysis. (JPG 164 kb)

Additional file 11: **Table S6.** League table presenting the results with restricted mean survival time difference (in month) at t* = 10 years and hazard ratio of the network meta-analysis (random effects, lower triangle) and of the conventional meta-analysis (random effects, upper triangle) for loco-regional control. (DOCX 17 kb)

Additional file 12: **Figure S5.** Forest plot for loco-regional control with hazard ratios (on the left) and restricted mean survival time difference at t* = 10 years (on the right) showing results from direct comparisons (random effects meta-analysis) and network meta-analysis. (JPG 172 kb)

## Abbreviations

AC: Adjuvant chemotherapy
CRT: Concomitant chemoradiotherapy
HR: Hazard ratio
IC: Induction chemotherapy
IPD: Individual patient data
LRC: Loco-regional control
NMA: Network meta-analysis
OS: Overall survival
PFS: Progression-free survival
RMST: Restricted mean survival time
rmstD: Restricted mean survival time difference
RT: Radiotherapy
