# Supplementary figures and images for: Individual patient data network meta-analysis using either restricted mean survival time difference or hazard ratios: is there a difference? A case study on locoregionally advanced nasopharyngeal carcinomas
Source: Syst Rev. 2019 Apr 15;8:96. doi: 10.1186/s13643-019-0984-x (PMC6463649; doi:10.1186/s13643-019-0984-x)

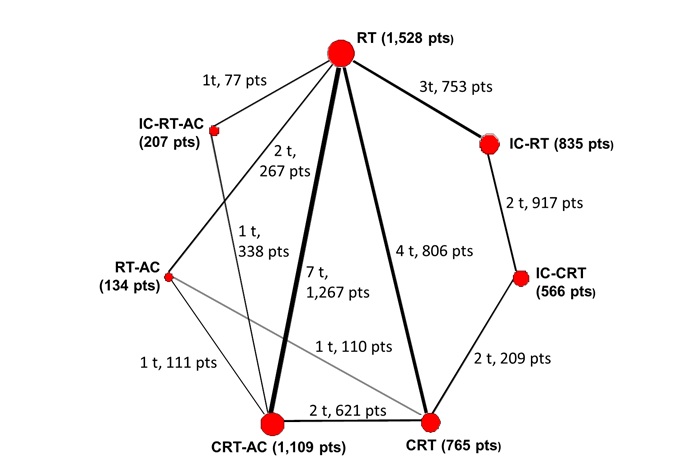

Supplement: Supplementary file 1 — Figure S1. Graphical representation of the trial network for overall survival. (JPG 51 kb) [file 13643_2019_984_MOESM1_ESM.jpg]

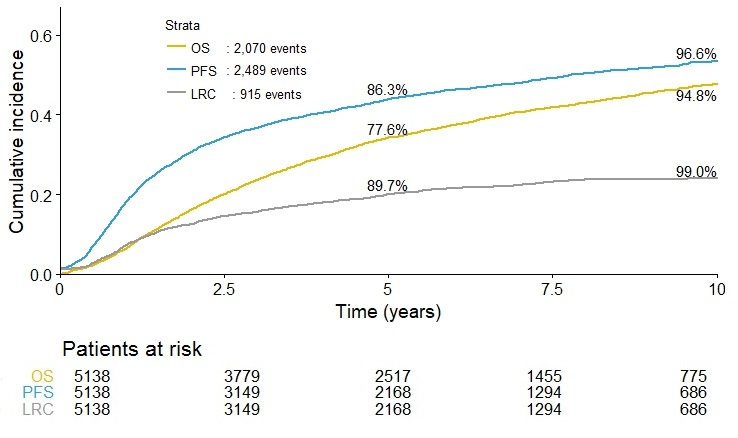

Supplement: Supplementary file 2 — Figure S2. Cumulative incidence curves for overall survival, progression free survival and loco-regional control events. (TIF 179 kb) [file 13643_2019_984_MOESM2_ESM.tif]

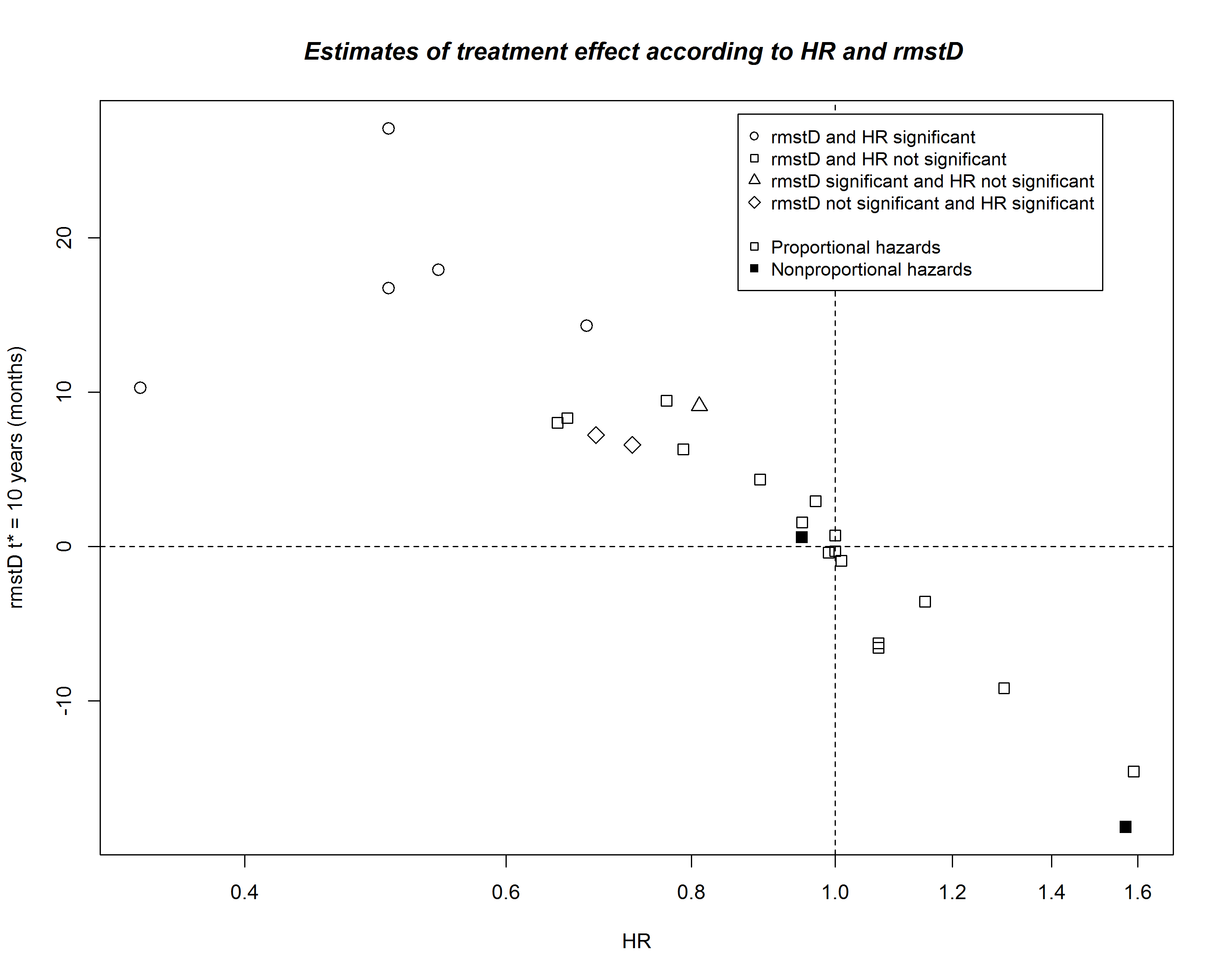

Supplement: Supplementary file 4 — Figure S3. Estimates of treatment effect for overall survival according to hazard ratios and restricted mean survival time difference at t* = 10 years. (TIFF 89 kb) [file 13643_2019_984_MOESM4_ESM.tiff]

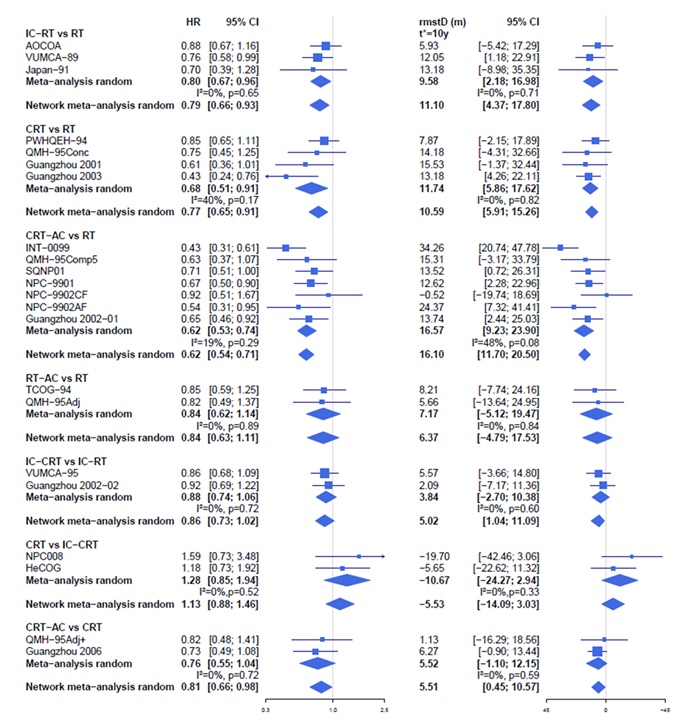

Supplement: Supplementary file 10 — Figure S4. Forest plot for progression-free survival with hazard ratios (on the left) and restricted mean survival time difference at t* = 10 years (on the right) showing results from direct comparisons (random effects meta-analysis) and network meta-analysis. (JPG 164 kb) [file 13643_2019_984_MOESM10_ESM.jpg]

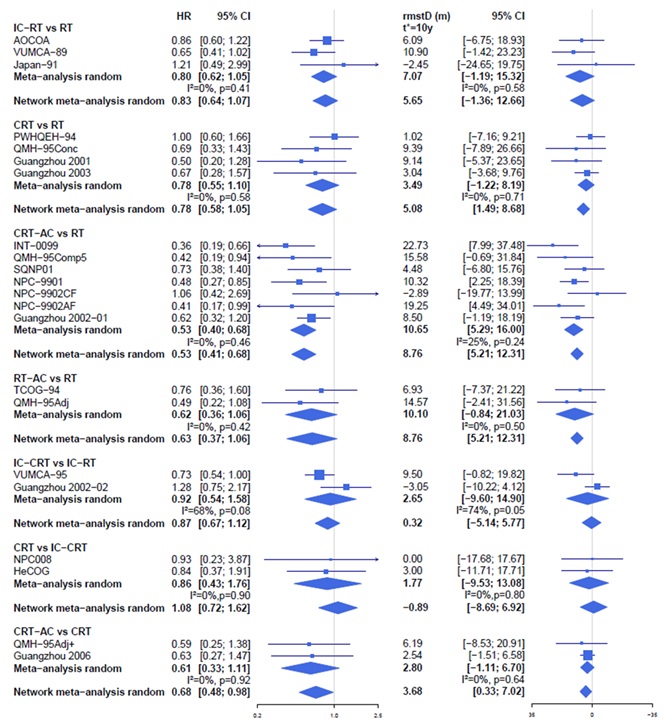

Supplement: Supplementary file 12 — Figure S5. Forest plot for loco-regional control with hazard ratios (on the left) and restricted mean survival time difference at t* = 10 years (on the right) showing results from direct comparisons (random effects meta-analysis) and network meta-analysis. (JPG 172 kb) [file 13643_2019_984_MOESM12_ESM.jpg]
