# Supplementary material for: Individual patient data network meta-analysis using either restricted mean survival time difference or hazard ratios: is there a difference? A case study on locoregionally advanced nasopharyngeal carcinomas
Source: Syst Rev. 2019 Apr 15;8:96. doi: 10.1186/s13643-019-0984-x (PMC6463649; doi:10.1186/s13643-019-0984-x)
Supplement: Supplementary file 3 — Table S1. Descriptive table of RMST per study and per arm and the rmstD at t* = 5 years and t* = 10 years for overall survival, expressed in months, and follow-up information. (DOCX 33 kb) [file 13643_2019_984_MOESM3_ESM.docx]

**Additional file 3: Table S1.** Descriptive table of RMST per study and per arm and the rmstD at t*= 5 years and t*= 10 years for overall survival, expressed in months, and follow-up information.

| Trial comparison | Control arm Experimental arm | N of event / N of patient | RMST t*= 5 years | rmstD t*= 5 years | RMST t*= 10 years | rmstD t*= 10 years | Latest event time (years) | Median follow-up (years) |  |
| --- | --- | --- | --- | --- | --- | --- | --- | --- | --- |
| AOCOA | RT | 55/167 | 46.1 | 0.66 | 79.4 | -0.39 | 12.9 | 5.4 |  |
|  | IC-RT | 54/167 | 46.8 |  | 79.0 |  | 12.8 |  |  |
| VUMCA-89 | RT | 93/168 | 38.5 | 0.56 | 64.0 | 0.73 | 11.8 | 7.0 |  |
|  | IC-RT | 94/171 | 39.1 |  | 64.7 |  | 11.8 |  |  |
| Japan-91 | RT | 20/40 | 47.6 | 0.91 | 72.0 | 9.44 | 9.2 | 6.2* |  |
|  | IC-RT | 17/40 | 48.5 |  | 81.4 |  | 8.8 |  |  |
| PWHQEH-94 | RT | 101/176 | 47.3 | 4.24 | 80.3 | 9.11 | 17.2 | 14.1 |  |
|  | CRT | 92/174 | 51.5 |  | 89.5 |  | 17.9 |  |  |
| QMH-95Conc‡ | RT | 24/55 | 52.6 | 1.18 | 94.1 | -0.30 | 17.3 | 14.0 |  |
|  | CRT | 25/56 | 53.7 |  | 93.8 |  | 17.5 |  |  |
| Guangzhou 2001 | RT | 31/56 | 45.7 | 7.90 | 76.4 | 17.94 | 11.4 | 9.6 |  |
|  | CRT | 21/59 | 53.6 |  | 94.4 |  | 11.4 |  |  |
| Guangzhou 2003 | RT | 26/114 | 57.2 | 1.95 | 104.1 | 10.29 | 9.8 | 7.6* |  |
|  | CRT | 9/116 | 59.1 |  | 114.4 |  | 9.8 |  |  |
| INT-0099 | RT | 79/96 | 36.5 | 11.92 | 55.9 | 27.08 | 20.4 | 16.8 |  |
|  | CRT-AC | 59/97 | 48.4 |  | 82.9 |  | 21.7 |  |  |
| QMH-95Comp5‡^ | RT | 24/55 | 52.6 | 1.79 | 94.1 | 8.02 | 17.3 | 14.0 |  |
|  | CRT-AC | 19/57 | 54.3 |  | 102.2 |  | 17.2 |  |  |
| SQNP01 | RT | 72/110 | 44.7 | 5.30 | 70.6 | 14.30 | 14.5 | 11.9 |  |
|  | CRT-AC | 60/111 | 50.0 |  | 84.9 |  | 15.1 |  |  |
| NPC-9901 | RT | 93/176 | 47.7 | 1.50 | 80.9 | 6.58 | 12.5 | 10.4 |  |
|  | CRT-AC | 69/172 | 49.2 |  | 87.5 |  | 13.1 |  |  |
| NPC-9902CF | RT | 18/42 | 50.9 | 1.67 | 87.6 | 2.94 | 12.6 | 10.6 |  |
|  | CRT-AC | 22/51 | 52.5 |  | 90.5 |  | 13.2 |  |  |
| NPC-9902AF | RT | 29/52 | 49.4 | 6.37 | 82.4 | 16.73 | 12.6 | 10.6 |  |
|  | CRT-AC | 15/44 | 55.8 |  | 99.1 |  | 12.8 |  |  |
| Guangzhou 2002-01 | RT | 65/158 | 47.6 | 4.68 | 80.6 | 7.21 | 8.4 | 6.2* |  |
|  | CRT-AC | 52/158 | 52.2 |  | 87.8 |  | 8.4 |  |  |
| TCOG-94 | RT | 53/78 | 44.8 | -1.52 | 72.9 | 1.57 | 17.7 | 15.0 |  |
|  | RT-AC | 52/80 | 43.3 |  | 74.4 |  | 18.4 |  |  |
| QMH-95Adj‡ | RT | 24/55 | 52.6 | -3.24 | 94.1 | -6.55 | 17.3 | 14.0 |  |
|  | RT-AC | 24/54 | 49.3 |  | 87.6 |  | 17.4 |  |  |
| VUMCA-95 | IC-RT | 116/253 | 42.7 | 0.67 | 67.3 | 4.33 | 9.1 | 5.8* |  |
|  | IC-CRT | 111/256 | 43.4 |  | 71.6 |  | 9.1 |  |  |
| Guangzhou 2002-02 | IC-RT | 81/204 | 51.4 | -1.63 | 86.8 | 0.60 | 9.2 | 7.4* |  |
|  | IC-CRT | 73/204 | 49.7 |  | 87.4 |  | 8.8 |  |  |
| NPC008 | CRT | 14/31 | 46.3 | 9.82 | 79.9 | 18.16 | 9.5 | 8.4* |  |
|  | IC-CRT | 12/34 | 56.1 |  | 98.1 |  | 9.7 |  |  |
| HeCOG | CRT | 29/72 | 45.1 | 0.03 | 78.8 | 0.91 | 8.2 | 6.7* |  |
|  | IC-CRT | 29/72 | 45.1 |  | 79.7 |  | 8.4 |  |  |
| QMH-95Adj+‡ | CRT | 25/56 | 53.7 | 0.61 | 93.8 | 8.32 | 17.5 | 14.0 |  |
|  | CRT-AC | 19/57 | 54.3 |  | 102.2 |  | 17.2 |  |  |
| Guangzhou 2006 | CRT | 34/257 | 54.0 | 1.53 | 93.7 | 6.30 | 5.0 | 3.2* |  |
|  | CRT-AC | 26/251 | 55.5 |  | 100.0 |  | 5.0 |  |  |
| QMH-95Conc+‡ | RT-AC | 24/54 | 49.3 | 5.03 | 87.6 | 14.56 | 17.4 | 14.0 |  |
|  | CRT-AC | 19/57 | 54.3 |  | 102.2 |  | 17.2 |  |  |
| QMH-95Comp6‡^ | RT-AC | 24/54 | 49.3 | 4.42 | 87.6 | 6.25 | 17.4 | 14.0 |  |
|  | CRT | 25/56 | 53.7 |  | 93.8 |  | 17.5 |  |  |
| PWH-88 | RT | 13/40 | 46.5 | -3.28 | 69.0 | -9.17 | 6.0 | 2.9* | |
|  | IC-RT-AC | 15/37 | 43.2 |  | 59.8 |  | 5.9 |  |  |
| Shanghai 2004 | IC-RT-AC | 39/167 | 56.0 | 0.32 | 93.8 | 3.57 | 7.7 | 5.6* |  |
|  | CRT-AC | 35/165 | 56.3 |  | 97.4 |  | 7.6 |  |  |

* Not taking into account survival extrapolation until 10 year by the Brown et al method^16^.

AC, adjuvant chemotherapy; CRT, concomitant chemoradiotherapy; IC, induction chemotherapy; RMST, restricted mean survival time; rmstD, restricted mean survival time difference; RT, radiotherapy.

‡One 2 x 2 factorial design trial QMH-95 was analyzed as a multiarm trial and split into six comparisons for proper modeling in the netmeta package.

^ comparison estimated using individual patient data, required for computation of multi-arms trials.
