# Supplementary material for: Individual patient data network meta-analysis using either restricted mean survival time difference or hazard ratios: is there a difference? A case study on locoregionally advanced nasopharyngeal carcinomas
Source: Syst Rev. 2019 Apr 15;8:96. doi: 10.1186/s13643-019-0984-x (PMC6463649; doi:10.1186/s13643-019-0984-x)
Supplement: Supplementary file 5 — Table S2. League table presenting the results with hazard ratio of the network meta-analysis (random effects, lower triangle) and of the conventional meta-analysis (random effects, upper triangle) for overall survival. (DOCX 18 kb) [file 13643_2019_984_MOESM5_ESM.docx]

**Additional file 5: Table S2.** League table presenting the results with hazard ratio of the network meta-analysis (random effects, lower triangle) and of the conventional meta-analysis (random effects, upper triangle) for overall survival.

*I²=5.5%, heterogeneity (within design) p=0.30, inconsistency (between designs) p=0.54. Individual trial (comparison) HR are given in Ribassin et al (Supplementary Table 11)*^14^

*As a convention the cells contain the hazard ratio (HR; 95% confidence interval) of the treatment with the higher number compared to the treatment with the lower number. For example the cell that joins treatments 4 (CRT) and 5 (CRT-AC) gives the HR of treatment 5 vs. 4 (CRT-AC vs. CRT).*

| RT (1) | 0.97  [0.78; 1.20] |  | 0.65 [0.44; 0.97] | 0.65 [0.56; 0.76] | 0.99 [0.72; 1.36] | 1.30* [0.62; 2.73] |
| --- | --- | --- | --- | --- | --- | --- |
| 0.92 [0.74; 1.13] | IC-RT (2) | 0.91 [0.75; 1.12] |  |  |  |  |
| 0.80 [0.62; 1.04] | 0.87 [0.72; 1.07] | IC-CRT (3) | 1.15 [0.75; 1.77] |  |  |  |
| 0.77 [0.63; 0.93] | 0.84 [0.65; 1.08] | 0.96 [0.72; 1.27] | CRT (4) | 0.73 [0.50; 1.07] | 1.07* [0.61; 1.87] |  |
| 0.65 [0.56; 0.76] | 0.71 [0.55; 0.92] | 0.81  [0.61; 1.09] | 0.85 [0.68; 1.07] | CRT-AC (5) | 1.59* [0.87; 2.91] | 1.15* [0.73; 1.81] |
| 0.96 [0.70; 1.30] | 1.04 [0.72; 1.51] | 1.19 [0.80; 1.78] | 1.25 [0.89; 1.76] | 1.47 [1.05; 2.05] | RT-AC (6) |  |
| 0.87 [0.58; 1.31] | 0.95 [0.60; 1.51] | 1.09 [0.67; 1.77] | 1.14 [0.73; 1.78] | 1.34 [0.90; 1.99] | 0.91 [0.55; 1.52] | IC-RT-AC (7) |

|  | same direction of treatment effect but difference in significance between HR and rmstD |
| --- | --- |
|  | different direction of treatment effect but both HR and rmstD are not significant |

*RT= radiotherapy, IC= induction chemotherapy, CRT= concomitant chemo-radiotherapy, AC= adjuvant chemotherapy, * comparison with only one trial*

*Note: This league table is slightly different from the one previously published*^14^ *due to the use of random effect NMA.*
