# Supplementary material for: Individual patient data network meta-analysis using either restricted mean survival time difference or hazard ratios: is there a difference? A case study on locoregionally advanced nasopharyngeal carcinomas
Source: Syst Rev. 2019 Apr 15;8:96. doi: 10.1186/s13643-019-0984-x (PMC6463649; doi:10.1186/s13643-019-0984-x)
Supplement: Supplementary file 6 — Text S1. Details of differences between restricted mean survival time difference and hazard ratio in the network meta-analysis for overall survival. Text S2 Details of differences between restricted mean survival time difference and hazard ratio in the network meta-analysis for progression-free survival. Text S3 Details of differences between restricted mean survival time difference and hazard ratio in the network meta-analysis for loco-regional control. (DOCX 25 kb) [file 13643_2019_984_MOESM6_ESM.docx]

**Additional file 6: Text 1:** Details of differences between restricted mean survival time difference and hazard ratio in the network meta-analysis for overall survival.

For the difference in the comparison of IC-CRT with RT, where HR was non-significant, 0.80 (95% CI, 0.62 to 1.04), while rmstD(t*= 10 years) was significantly in favor of IC-CRT, with a value of 8.71 months (95% CI, 0.26 to 17.16): two trials for which the use of rmstD instead of HR was more in favor of IC-CRT can explain this difference: in the comparison of CRT with IC-CRT, NPC008^32^ had non-proportional hazards; and in the comparison of CRT with RT, PWHQEH-94^42^ had a non-significant HR while rmstD was significantly in favor of CRT.

For the difference in the comparison of CRT-AC with IC-RT, where HR was significantly in favor of CRT-AC, 0.71 (95% CI, 0.55 to 0.92), while rmstD(t*= 10 years) was not, 7.58 months (95% CI, -0.68 to 15.84): two trials can explain this difference: in the comparison of CRT-AC with RT, NPC-9901^29^ and Guangzhou 2002-01^34^ had a significant HR in favor of CRT-AC while rmstD was non-significant.

It may also be noted that the two trials with non-proportional hazards (NPC008^32^ and Guangzhou 2002-02^33^) are involved in the two pairwise comparisons with a change in statistical significance.

**Text 2:** Details of differences between restricted mean survival time difference and hazard ratio in the network meta-analysis for progression-free survival.

Three had a different direction of treatment effect (HR < 1 and rmstD < 0) but both HR and rmstD were not significant. For the comparison of CRT-AC with IC-RT with only indirect information, the direction of treatment effect was the same but the significance changed: HR was significant and equaled to 0.79 (95% CI, 0.64 to 0.98), and rmstD(t*= 10 years) was non-significant with a value of 5.00 months (95% CI, -2.83 to 12.83). Two trials with non-proportional hazards were located in the comparison used to perform this indirect comparison (CRT-AC vs. RT: Int-0099^25^ and NPC-9902AF^30^). The reversal in the ranking (IC-RT becomes better than CRT with rmstD) was partially explained by the change in the direction of treatment effect in the comparison of CRT with IC-RT where HR was 0.98 (95% CI, 0.78 to 1.22) while rmstD(t*= 10 years) was -0.51 months (95% CI, -8.15 to 7.13).

**Text 3:** Details of differences between restricted mean survival time difference and hazard ratio in the network meta-analysis for loco-regional control.

Two had a different direction of treatment effect but both HR and rmstD were not significant. And two had the same direction of treatment effect but changed the significance. The first one concerned the pairwise comparison of CRT-AC with IC-RT with only indirect information: HR significant, 0.64 (95% CI, 0.45 to 0.91) and rmstD(t*= 10 years) non-significant, 3.11 months (95% CI, -4.47 to 10.69). The three trials with non-proportional hazards were located in the two direct comparisons used to perform this indirect comparison (CRT-AC vs RT: Int-0099^25^ and NPC-9902AF^30^; IC-RT vs RT: VUMCA-89^24^). There was also a discordance for the significance of HR and rmstD in the trial comparison of CRT-AC with RT-AC (QMH-95 Comp 5^27^). The second one concerned the pairwise comparison of CRT with RT: HR non-significant, 0.78 (95% CI, 0.58 to 1.05) and rmstD(t*= 10 years) significant, 5.08 months (95% CI, 1.49 to 8.68). Here, there was direct information to support the result. The network effect decreased the uncertainty of the treatment effect estimates for both HR and rmstD, while it also increased the treatment effect size for rmstD: this led to a significant rmstD.

See the paper for the references
