# Supplementary material for: Individual patient data network meta-analysis using either restricted mean survival time difference or hazard ratios: is there a difference? A case study on locoregionally advanced nasopharyngeal carcinomas
Source: Syst Rev. 2019 Apr 15;8:96. doi: 10.1186/s13643-019-0984-x (PMC6463649; doi:10.1186/s13643-019-0984-x)
Supplement: Supplementary file 7 — Table S3. League table presenting the results with difference in restricted mean survival time (in month) and hazard ratio with censor at 5 years of the network meta-analysis (random effects, lower triangle) and of the conventional meta-analysis (random effects, upper triangle) for overall survival at t* = 5 years (sensitivity analysis). (DOCX 17 kb) [file 13643_2019_984_MOESM7_ESM.docx]

**Additional file 7: Table S3.** League table presenting the results with difference in restricted mean survival time (in month) and hazard ratio with censor at 5 years of the network meta-analysis (random effects, lower triangle) and of the conventional meta-analysis (random effects, upper triangle) for overall survival at t* = 5 years (sensitivity analysis).

*As a convention the cells contain the difference in restricted mean survival time in month (rmstD; 95% confidence interval) of the treatment with the higher number compared to the treatment with the lower number. For example the cell that joins treatments 4 (CRT) and 5 (CRT-AC) gives the rmstD of treatment 5 vs. 4 (CRT-AC vs. CRT).*

*Difference in restricted mean survival time: I²=35.9%, heterogeneity (within design) p=0.07, inconsistency (between designs) p=0.24. Individual trial (comparison) rmstD are given in Supplementary Table 1.*

| RT (1) | 0.67 [-2.40; 3.73] |  | 3.16 [0.88; 5.44] | 4.56 [2.05; 7.07] | -2.40 [-6.90; 2.10] | -3.28* [-12.56; 6.01] |
| --- | --- | --- | --- | --- | --- | --- |
| 2.38 [-0.84; 5.59] | IC-RT (2) | -0.65 [-3.08; 1.78] |  |  |  |  |
| 3.26 [-0.56; 7.09] | 0.89 [-2.15; 3.93] | IC-CRT (3) | -4.82 [-14.42; 4.77] |  |  |  |
| 2.66 [0.58; 4.75] | 0.29 [-3.27; 3.85] | -0.60 [-4.49; 3.29] | CRT (4) | 1.35 [-0.78; 3.49] | -4.42* [-10.23; 1.40] |  |
| 4.20 [2.23; 6.17] | 1.83 [-1.87; 5.52] | 0.94 [-3.24; 5.11] | 1.54 [-0.87; 3.94] | CRT-AC (5) | -5.03* [-10.94; 0.88] | -0.32* [-2.41; 1.78] |
| -1.72 [-6.31; 2.88] | -4.09 [-9.64; 1.46] | -4.98 [-10.86; 0.91] | -4.38 [-9.17; 0.41] | -5.92 [-10.69; -1.14] | RT-AC (6) |  |
| 2.92 [-1.09; 6.92] | 0.54 [-4.55; 5.63] | -0.35 [-5.80; 5.10] | 0.25 [-4.02; 4.52] | -1.29 [-4.92; 2.34] | 4.63 [-1.30; 10.56] | IC-RT-AC (7) |

*Hazard ratio: I²=21.1%, heterogeneity (within design) p=0.25, inconsistency (between designs) p=0.25.*

| RT (1) | 0.94 [0.75; 1.17] |  | 0.62 [0.43; 0.88] | 0.63 [0.53; 0.76] | 1.08 [0.68; 1.70] | 1.28* [0.59; 2.76] |
| --- | --- | --- | --- | --- | --- | --- |
| 0.85 [0.66; 1.09] | IC-RT (2) | 0.98 [0.79; 1.22] |  |  |  |  |
| 0.76 [0.55; 1.05] | 0.89 [0.69; 1.16] | IC-CRT (3) | 1.65 [0.49; 5.64] |  |  |  |
| 0.72 [0.56; 0.92] | 0.85 [0.61; 1.17] | 0.95 [0.67; 1.35] | CRT (4) | 0.77 [0.50; 1.18] | 1.45* [0.71; 2.96] |  |
| 0.62 [0.51; 0.76] | 0.74 [0.54; 1.01] | 0.83 [0.57; 1.20] | 0.87 [0.65; 1.16] | CRT-AC (5) | 1.95* [0.92; 4.16] | 1.19* [0.72; 1.96] |
| 1.05 [0.70; 1.56] | 1.24 [0.77; 1.98] | 1.38 [0.83; 2.30] | 1.46 [0.93; 2.28] | 1.67 [1.09; 2.58] | RT-AC (6) |  |
| 0.88 [0.55; 1.43] | 1.05 [0.61; 1.80] | 1.17 [0.66; 2.08] | 1.23 [0.72; 2.09] | 1.41 [0.89; 2.26] | 0.85 [0.46; 1.57] | IC-RT-AC (7) |

|  | different direction of treatment effect but both HR and rmstD are not significant |
| --- | --- |

*RT= radiotherapy, IC= induction chemotherapy, CRT= concomitant chemo-radiotherapy, AC= adjuvant chemotherapy, * comparison with only one trial*
