# Supplementary material for: Individual patient data network meta-analysis using either restricted mean survival time difference or hazard ratios: is there a difference? A case study on locoregionally advanced nasopharyngeal carcinomas
Source: Syst Rev. 2019 Apr 15;8:96. doi: 10.1186/s13643-019-0984-x (PMC6463649; doi:10.1186/s13643-019-0984-x)
Supplement: Supplementary file 8 — Table S4. League tables presenting the results with difference in restricted mean survival time (in month) at t* = 10 years and hazard ratio of the network meta-analysis (random effects, lower triangle) and of the conventional meta-analysis (random effects, upper triangle) for overall survival after exclusion of NPC008 and Guangzhou 2002-02 trials which had significant test for non-proportionality (sensitivity analysis). (DOCX 17 kb) [file 13643_2019_984_MOESM8_ESM.docx]

**Additional file 8: Table S4.** League tables presenting the results with difference in restricted mean survival time (in month) at t* = 10 years and hazard ratio of the network meta-analysis (random effects, lower triangle) and of the conventional meta-analysis (random effects, upper triangle) for overall survival after exclusion of NPC008 and Guangzhou 2002-02 trials which had significant test for non-proportionality (sensitivity analysis).

*As a convention the cells contain the difference in restricted mean survival time in month (rmstD; 95% confidence interval) of the treatment with the higher number compared to the treatment with the lower number. For example the cell that joins treatments 4 (CRT) and 5 (CRT-AC) gives the rmstD/HR of treatment 5 vs. 4 (CRT-AC vs. CRT).*

*Difference in restricted mean survival time: I²=3.3%, heterogeneity (within design) p=0.29, inconsistency (between designs) p=0.59.*

| RT (1) | 1.52 [-5.85; 8.89] |  | 9.56 [4.82; 14.30] | 11.67 [5.82; 17.52] | -2.17 [-12.87; 8.53] | -9.17* [-32.69; 14.36] |
| --- | --- | --- | --- | --- | --- | --- |
| 2.05 [-4.94; 9.03] | IC-RT (2) | 4.33* [-5.07; 13.73] |  |  |  |  |
| 7.20 [-2.66; 17.07] | 5.16 [-3.38; 13.70] | IC-CRT (3) | -0.91* [-16.86; 15.04] |  |  |  |
| 8.61 [4.27; 12.94] | 6.56 [-1.34; 14.46] | 1.40 [-8.72; 11.52] | CRT (4) | 6.96 [-0.84; 14.76] | -6.25* [-21.66; 9.16] |  |
| 12.06 [7.87; 16.26] | 10.02 [1.94; 18.09] | 4.86 [-5.71; 15.43] | 3.46 [-1.81; 8.72] | CRT-AC (5) | -14.56* [-29.53; 0.40] | -3.57* [-12.52; 5.39] |
| -0.48 [-10.38; 9.43] | -2.52 [-14.60; 9.55] | -7.68 [-21.55; 6.18] | -9.08 [-19.49; 1.32] | -12.54 [-22.87; -2.21] | RT-AC (6) |  |
| 6.16 [-3.17; 15.49] | 4.12 [-7.50; 15.73] | -1.05 [-14.52; 12.43] | -2.45 [-12.36; 7.47] | -5.90 [-14.51; 2.71] | 6.64 [-6.68; 19.96] | IC-RT-AC (7) |

*Hazard ratio: I²=6.4%, heterogeneity (within design) p=0.21, inconsistency (between designs) p=0.67.*

| RT (1) | 0.97 [0.78; 1.20] |  | 0.65 [0.44; 0.97] | 0.65 [0.56; 0.76] | 0.99 [0.72; 1.36] | 1.30* [0.62; 2.73] |
| --- | --- | --- | --- | --- | --- | --- |
| 0.94 [0.76; 1.17] | IC-RT (2) | 0.89* [0.69; 1.16] |  |  |  |  |
| 0.82 [0.60; 1.11] | 0.87 [0.67; 1.12] | IC-CRT (3) | 1.01* [0.60; 1.68] |  |  |  |
| 0.75 [0.61; 0.91] | 0.79 [0.60; 1.04] | 0.91 [0.66; 1.26] | CRT (4) | 0.73 [0.50; 1.07] | 1.07* [0.61; 1.87] |  |
| 0.65 [0.56; 0.75] | 0.69 [0.53; 0.89] | 0.79 [0.57; 1.11] | 0.87 [0.69; 1.10] | CRT-AC (5) | 1.59* [0.87; 2.91] | 1.15* [0.73; 1.81] |
| 0.95 [0.70; 1.30] | 1.01 [0.69; 1.46] | 1.16 [0.76; 1.78] | 1.28 [0.90; 1.81] | 1.47 [1.05; 2.05] | RT-AC (6) |  |
| 0.87 [0.58; 1.31] | 0.92 [0.58; 1.46] | 1.06 [0.64; 1.77] | 1.17 [0.74; 1.83] | 1.34 [0.90; 2.00] | 0.91 [0.55; 1.52] | IC-RT-AC (7) |

|  | different direction of treatment effect but both HR and rmstD are not significant |
| --- | --- |

*RT= radiotherapy, IC= induction chemotherapy, CRT= concomitant chemo-radiotherapy, AC= adjuvant chemotherapy, * comparison with only one trial*
