# Supplementary material for: Individual patient data network meta-analysis using either restricted mean survival time difference or hazard ratios: is there a difference? A case study on locoregionally advanced nasopharyngeal carcinomas
Source: Syst Rev. 2019 Apr 15;8:96. doi: 10.1186/s13643-019-0984-x (PMC6463649; doi:10.1186/s13643-019-0984-x)
Supplement: Supplementary file 9 — Table S5. League tables presenting the results with restricted mean survival time difference (in month) at t* = 10 years and hazard ratio of the network meta-analysis (random effects, lower triangle) and of the conventional meta-analysis (random effects, upper triangle) for progression-free survival. (DOCX 20 kb) [file 13643_2019_984_MOESM9_ESM.docx]

**Additional file 9: Table S5.** League tables presenting the results with restricted mean survival time difference (in month) at t* = 10 years and hazard ratio of the network meta-analysis (random effects, lower triangle) and of the conventional meta-analysis (random effects, upper triangle) for progression-free survival.

*As a convention the cells contain the difference in restricted mean survival time in month (rmstD; 95% confidence interval) of the treatment with the higher number compared to the treatment with the lower number. For example the cell that joins treatments 4 (CRT) and 5 (CRT-AC) gives the rmstD of treatment 5 vs. 4 (CRT-AC vs. CRT).*

*Difference in restricted mean survival time: I²=0%, heterogeneity (within design) p=0.22, inconsistency (between designs) p=0.86. Individual trial (comparison) HR are given in Ribassin et al (Supplementary Table 12)*^14^

| RT (1) | 9.58 [2.18; 16.98] |  | 11.74 [5.86; 17.62] | 16.57 [9.23; 23.90] | 7.17 [-5.12; 19.47] | -3.79* [-27.24; 19.65] |
| --- | --- | --- | --- | --- | --- | --- |
| 11.10 [4.37; 17.80] | IC-RT (2) | 3.84 [-2.70; 10.38] |  |  |  |  |
| 16.12 [7.97; 24.27] | 5.02 [-1.04; 11.09] | IC-CRT (3) | -10.67 [-24.27; 2.94] |  |  |  |
| 10.59 [5.91; 15.26] | -0.51 [-8.15; 7.13] | -5.53 [-14.09; 3.03] | CRT (4) | 5.52 [-1.10; 12.15] | -8.52* [-26.80; 9.77] |  |
| 16.10 [11.70; 20.50] | 5.00 [-2.83; 12.83] | -0.02 [-8.99; 8.95] | 5.51 [0.45; 10.57] | CRT-AC (5) | -9.65* [-27.93; 8.63] | -5.79* [-15.88; 4.31] |
| 6.37 [-4.79; 17.53] | -4.73 [-17.67; 8.21] | -9.75 [-23.43; 3.93] | -4.22 [-15.86; 7.42] | -9.73 [-21.30; 1.84] | RT-AC (6) |  |
| 8.10 [-1.88; 18.09] | -2.99 [-14.91; 8.93] | -8.02 [-20.73; 4.70] | -2.48 [-12.90; 7.93] | -7.99 [-17.29; 1.31] | 1.74 [-12.96; 16.43] | IC-RT-AC (7) |

*Hazard ratio: I²=0%, heterogeneity (within design) p=0.25, inconsistency (between designs) p=0.96. Individual trial (comparison) HR are given in Ribassin et al (Supplementary Table 12)*^14^

| RT (1) | 0.80 [0.67; 0.96] |  | 0.68 [0.51; 0.91] | 0.62 [0.53; 0.74] | 0.84 [0.62; 1.14] | 0.99* [0.51; 1.95] |
| --- | --- | --- | --- | --- | --- | --- |
| 0.79 [0.66; 0.93] | IC-RT (2) | 0.88 [0.74; 1.06] |  |  |  |  |
| 0.68 [0.54; 0.85] | 0.86 [0.73; 1.02] | IC-CRT (3) | 1.28 [0.85; 1.94] |  |  |  |
| 0.77 [0.65; 0.91] | 0.98 [0.78; 1.22] | 1.13 [0.88; 1.46] | CRT (4) | 0.76 [0.55; 1.04] | 1.07* [0.63; 1.82] |  |
| 0.62 [0.54; 0.71] | 0.79 [0.64; 0.98] | 0.92 [0.71; 1.18] | 0.81 [0.66; 0.98] | CRT-AC (5) | 1.30* [0.75; 2.24] | 1.26* [0.86; 1.84] |
| 0.84 [0.63; 1.11] | 1.07 [0.77; 1.49] | 1.24 [0.86; 1.77] | 1.09 [0.79; 1.50] | 1.35 [0.99; 1.84] | RT-AC (6) |  |
| 0.83 [0.58; 1.17] | 1.05 [0.72; 1.55] | 1.22 [0.81; 1.84] | 1.08 [0.74; 1.57] | 1.33 [0.96; 1.86] | 0.99 [0.63; 1.54] | IC-RT-AC (7) |

|  | same direction of treatment effect but difference in significance between HR and rmstD |
| --- | --- |
|  | different direction of treatment effect but both HR and rmstD are not significant |

*RT= radiotherapy, IC= induction chemotherapy, CRT= concomitant chemo-radiotherapy, AC= adjuvant chemotherapy, * comparison with only one trial*
