# Supplementary material for: Individual patient data network meta-analysis using either restricted mean survival time difference or hazard ratios: is there a difference? A case study on locoregionally advanced nasopharyngeal carcinomas
Source: Syst Rev. 2019 Apr 15;8:96. doi: 10.1186/s13643-019-0984-x (PMC6463649; doi:10.1186/s13643-019-0984-x)
Supplement: Supplementary file 11 — Table S6. League table presenting the results with restricted mean survival time difference (in month) at t* = 10 years and hazard ratio of the network meta-analysis (random effects, lower triangle) and of the conventional meta-analysis (random effects, upper triangle) for loco-regional control. (DOCX 17 kb) [file 13643_2019_984_MOESM11_ESM.docx]

**Additional file 11: Table S6.** League table presenting the results with restricted mean survival time difference (in month) at t* = 10 years and hazard ratio of the network meta-analysis (random effects, lower triangle) and of the conventional meta-analysis (random effects, upper triangle) for loco-regional control.

*As a convention the cells contain the difference in restricted mean survival time in month (rmstD; 95% confidence interval) of the treatment with the higher number compared to the treatment with the lower number. For example the cell that joins treatments 4 (CRT) and 5 (CRT-AC) gives the HR of treatment 5 vs. 4 (CRT-AC vs. CRT).*

*Difference in restricted mean survival time: I²=0%, heterogeneity (within design) p=0.26, inconsistency (between designs) p=0.83.*

| RT (1) | 7.07 [-1.19; 15.32] |  | 3.49 [-1.22; 8.19] | 10.65 [5.29; 16.00] | 10.10 [-0.84; 21.03] | 5.81* [-16.94; 28.57] |
| --- | --- | --- | --- | --- | --- | --- |
| 5.65 [-1.36; 12.66] | IC-RT (2) | 2.65 [-9.60; 14.90] |  |  |  |  |
| 5.97 [-1.75; 13.68] | 0.32 [-5.14; 5.77] | IC-CRT (3) | 1.77 [-9.53; 13.08] |  |  |  |
| 5.08 [1.49; 8.68] | -0.57 [-7.95; 6.81] | -0.89 [-8.69; 6.92] | CRT (4) | 2.80 [-1.11; 6.70] | 5.19* [-10.33; 20.70] |  |
| 8.76 [5.21; 12.31] | 3.11 [-4.47; 10.69] | 2.79 [-5.32; 10.90] | 3.68 [0.33; 7.02] | CRT-AC (5) | -1.01* [-15.39; 13.38] | 4.17* [-4.45; 12.79] |
| 8.76 [-0.74; 18.27] | 3.11 [-8.59; 14.81] | 2.79 [-9.30; 14.88] | 3.68 [-6.06; 13.42] | 0.004 [-9.69; 9.70] | RT-AC (6) |  |
| 12.04 [3.40; 20.67] | 6.38 [-4.57; 17.34] | 6.07 [-5.27; 17.41] | 6.95 [-1.71; 15.61] | 3.28 [-4.79; 11.35] | 3.27 [-9.26; 15.81] | IC-RT-AC (7) |

*Hazard ratio: I²=0%, heterogeneity (within design) p=0.35, inconsistency (between designs) p=0.92.*

| RT (1) | 0.80 [0.62; 1.05] |  | 0.78 [0.55; 1.10] | 0.53 [0.40; 0.68] | 0.62 [0.36; 1.06] | 0.74* [0.20; 2.74] |
| --- | --- | --- | --- | --- | --- | --- |
| 0.83 [0.64; 1.07] | IC-RT (2) | 0.92 [0.54; 1.58] |  |  |  |  |
| 0.72 [0.51; 1.01] | 0.87 [0.67; 1.12] | IC-CRT (3) | 0.86 [0.43; 1.76] |  |  |  |
| 0.78 [0.58; 1.05] | 0.94 [0.65; 1.35] | 1.08 [0.72; 1.62] | CRT (4) | 0.61 [0.33; 1.11] | 0.69* [0.29; 1.61] |  |
| 0.53 [0.41; 0.68] | 0.64 [0.45; 0.91] | 0.74 [0.49; 1.12] | 0.68 [0.48; 0.98] | CRT-AC (5) | 1.16* [0.44; 3.11] | 0.82* [0.47; 1.43] |
| 0.63 [0.37; 1.06] | 0.76 [0.42; 1.35] | 0.87 [0.47; 1.62] | 0.81 [0.45; 1.43] | 1.18 [0.67; 2.09] | RT-AC (6) |  |
| 0.47 [0.27; 0.82] | 0.57 [0.31; 1.05] | 0.66 [0.35; 1.26] | 0.61 [0.33; 1.13] | 0.89 [0.53; 1.49] | 0.76 [0.36; 1.61] | IC-RT-AC (7) |

|  | same direction of treatment effect but difference in significance between HR and rmstD |
| --- | --- |
|  | different direction of treatment effect but both HR and rmstD are not significant |

*RT= radiotherapy, IC= induction chemotherapy, CRT= concomitant chemo-radiotherapy, AC= adjuvant chemotherapy, * comparison with only one trial*
